# Congenital Hemolytic Anemias: Is There a Role for the Immune System?

**DOI:** 10.3389/fimmu.2020.01309

**Published:** 2020-06-23

**Authors:** Anna Zaninoni, Elisa Fermo, Cristina Vercellati, Anna Paola Marcello, Wilma Barcellini, Paola Bianchi

**Affiliations:** UOS Fisiopatologia delle Anemie, UOC Ematologia, Fondazione IRCCS Ca' Granda Ospedale Maggiore Policlinico, Milan, Italy

**Keywords:** congenital hemolytic anemias, splenectomy, inflammation, cytokines, iron overload, naturally occurring antibodies

## Abstract

Congenital hemolytic anemias (CHAs) are a heterogeneous group of rare hereditary conditions including defects of erythrocyte membrane proteins, red cell enzymes, and disorders due to defective erythropoiesis. They are characterized by variable degree of anemia, chronic extravascular hemolysis, reduced erythrocyte life span, splenomegaly, jaundice, biliary lithiasis, and iron overload. Although few data are reported on the role of the immune system in CHAs, several immune-mediated mechanisms may be involved in the pathogenesis of these rare diseases. We reported in ~60% of patients with hereditary spherocytosis (HS), the presence of naturally-occurring autoantibodies (NAbs) directed against different membrane proteins (α- and β-spectrin, band 3, and dematin). Positive HS subjects showed a more hemolytic pattern and NAbs were more evident in aged erythrocytes. The latter is in line with the function of NAbs in the opsonization of damaged/senescent erythrocytes and their consequent removal in the spleen. Splenectomy, usually performed to reduce erythrocyte catheresis and improve Hb levels, has different efficacy in various CHAs. Median Hb increase is 3 g/dL in HS, 1.6–1.8 g/dL in pyruvate kinase deficiency (PKD), and 1 g/dL in congenital dyserythropoietic anemias (CDA) type II. Consistently with clinical severity, splenectomy is performed in 20% of HS, 45% of CDAII, and in 60% of PKD patients. Importantly, sepsis and thrombotic events have been registered, particularly in PKD with a frequency of ~7% for both. Furthermore, we analyzed the role of pro-inflammatory cytokines and found that interleukin 10 and interferon γ, and to a lesser extent interleukin 6, were increased in all CHAs compared with controls. Moreover, CDAII and enzymatic defects showed increased tumor necrosis factor-α and reduced interleukin 17. Finally, we reported that iron overload occurred in 31% of patients with membrane defects, in ~60% of CDAII cases, and in up to 82% of PKD patients (defined by MRI liver iron concentration >4 mg Fe/gdw). Hepcidin was slightly increased in CHAs compared with controls and positively correlated with ferritin and with the inflammatory cytokines interleukin 6 and interferon γ. Overall the results suggest the existence of a vicious circle between chronic hemolysis, inflammatory response, bone marrow dyserythropoiesis, and iron overload.

## Introduction

Congenital hemolytic anemias (CHAs) are a heterogeneous group of rare hereditary conditions characterized by reduced life span and premature removal of the erythrocytes from the circulation. They comprise defects of the erythrocyte membrane proteins and of red cell enzymes metabolism, as well as alterations at the level of erythrocyte precursors, resulting in defective bone marrow erythropoiesis. The typical examples of membrane defects are hereditary spherocytosis (HS), hereditary elliptocytosis (HE), and the group of hereditary stomatocytosis (HSt). Glucose-6-phosphate dehydrogenase (G6PD) and pyruvate kinase (PK), are the most common enzyme deficiencies, and congenital dyserythropoietic anemia (CDA) type II is the best studied form among defective erythropoiesis. The role of the immune system has been poorly investigated in these conditions, at variance with the several reports in hemoglobinopathies such as sickle cell disease and thalassemia, which are beyond the scope of this review.

In this review the role of naturally-occurring autoantibodies will be discussed focusing on their ability to opsonize damaged/senescent erythrocytes that are consequently removed in the spleen. Furthermore, as splenectomy is one of the therapeutic options in these conditions, we will describe the immunological abnormalities following this procedure, with particular reference to increased infectious and thrombotic risk. Finally, given the increasing interest in the occurrence of iron overload in CHAs and consequent relevant clinical complications, we will review available literature on this topic. We will focus on the pathophysiology of iron overload which is closely linked to inflammatory cytokines and to the hepcidin pathway, which in turn is straightly linked to the immune system.

## Clinical and Molecular Findings in CHAs

Although some hemolytic features are present also in hemoglobinopathies, the classic CHAs are characterized by chronic extravascular hemolysis, splenomegaly, jaundice, biliary lithiasis, and a variable degree of anemia and iron overload. The most relevant genetic basis of CHAs are shown in Table 1 and more detailed description of the different forms is given in the following sections.

**Table 1:** Genetic basis of congenital hemolytic anemias.

	**Gene**	**Gene location**	**Function**	**Trasmission**
**RBC membrane defects**				
*Hereditary spherocytosis*	*SPTA1*	1q23.1	Membrane skeletal network	AR
	*SPTB*	14q23.3	Membrane skeletal network	AD
	*SLC4A1*	17q21.31	Anion exchange channel Link to glycoltytic enzymes Vertical interactions	AD
	*ANK1*	8p11.21	Vertical interactions	AD, *de novo*
	*EPB42*	15q15.2	Stabilize band3/ankyrin complex	AR
*Hereditary elliptocyosis*	*SPTA1*	1q23.1	Membrane skeletal network	AD
	*SPTB*	14q23.3	Membrane skeletal network	AD
	*EPB41*	1p35.3	Stabilize spectrin-ankyrin contact	AD
*Hereditary pyropoikylocytosis*	*SPTA1/ SPTA1^*LELY*^* *SPTA1/ SPTB* *SPTB/SPTB*	1q23.1	Membrane skeletal network	AR
*Hereditary stomatocytosis*				
Dehydrated	*PIEZO1*	16q24.3	Mechanosensitive ion channel	AD
Overhydrated	*RHAG*	6p12.3	Rh -blood group	AD
Gardos Channelopathy	*KCNN4*	19q13.31	Potassium Ca2+-Activated Channel	AD, *de novo*
**RBC enzyme defects**				
Glucose-6-phosphate dehydrogenase deficiency	*G6PD*	Xq28	Hexose-monophosphate shunt	X-linked
Pyruvate kinase deficiency	*PK-LR*	1q22	Glycolysis	AR
Glucosephosphate isomerase deficiency	*GPI*	19q13.11	Glycolysis	AR
Triosephosphate isomerase deficiency	*TPI1*	12p13.31	Glycolysis	AR
Hexokinase deficiency	*HK1*	10q22.1	Glycolysis	AR
Phosphofructokinase deficiency	*PFK-M* *PFK-L*	12q13.31 21q22.3	Glycolysis	AR
Phosphoglycerate kinase deficiency	*PGK1*	Xq21.1	Glycolysis	X-linked
Pyrimidine-5′-nucleotidase deficiency	*NT5C3A*	7p14.3	Nucleotide metabolism	AR
Adenylate kinase deficiency	*AK1*	9q34.11	Nucleotide metabolism	AR
**Congenital dyserythropoietic anemias**				
CDAI	*CDAN1* *C15ORF41*	15q15.2 15q14	Microtubule attachments Restriction endonuclease	AR
CDAII	*SEC23B*	20p11.23	Vescicle trafficking	AR
CDAIII	*KIF23*	15q23	Cytokinesis	AD
CDA variants	*GATA1*	Xp11.23	Transcription factor	X-linked
	*KLF1*	19p13.13	Transcriptional activator	AD

*AR, Autosomic recessive; AD, Autosomic dominant*.

### Red Cell Membrane Disorders

Inherited RBC membrane disorders are caused by quantitative or qualitative defects in transmembrane or cytoskeletal proteins of erythrocytes (1–3). HS is the most frequent congenital hemolytic anemia in Caucasians, with reported prevalence ranging from 1:2,000 to 1:5,000, and is characterized by a highly heterogeneous molecular defect, involving the genes coding for RBC membrane proteins *SPTA1* (α-spectrin), *SPTB* (β- spectrin), *SLC4A1* (band 3), *ANK1* (ankyrin), *EPB42* (protein 4.2). In general, these abnormalities affect the vertical interactions between phospholipid bilayer and the cytoskeleton of RBC membrane, resulting in a progressive change of the discocytes into osmotically fragile spherocytes that are recognized and sequestered by the spleen (4). HE, characterized by the presence of elliptocytes in peripheral blood smear, is more prevalent in malaria endemic regions in West Africa; it is usually an asymptomatic condition, but moderate to severe anemia may be present in ~10% of cases (5). The severe recessive variant is hereditary pyropoikilocytosis, in which the significant membrane fragmentation and reduced surface area is mostly caused by a pathogenic mutation in *SPTA1* gene inherited *in trans* to the hypomorphic variant αLELY (Low Expression LYon) (6). In HSt the inability to regulate the cation homeostasis lead to inappropriate shrinkage (dehydrated HSt) or swelling (overhydrated HSt) of the RBCs (7–13). Finally, “Gardos cahnnelopathy” is a recently described form of HSt with some differences in the clinical phenotype and hematological features, caused by mutations in *KCNN4* gene (14–18).

### Defects of Red Cell Metabolism

CHAs also occur as a consequence of RBC metabolism defects, affecting one of the three main metabolic pathways: the Embden-Myerhof pathway (glycolysis), the nucleotide metabolism, and the exose-monophosphate shunt. G6PD deficiency is the most common erythroenzymopathy, usually causing acute hemolysis during oxidative stress, with the exception of the class-I variants, which also result in chronic hemolysis (19, 20). Among the abnormalities of glycolytic enzymes, the most common is PK deficiency (PKD) (21–25), followed by glucosephosphate isomerase and hexokinase deficiency (26–29). Pyrimidine 5′-nucleotidase is the most frequent defect of nucleotide metabolism (30), whereas adenylate kinase deficicency has been reported in 12 families only (31). When the involved gene is ubiquitously expressed, the enzymopathy may be associated to extra-hematological signs such neuromuscular abnormalities, myopathy and mental retardation, as in the case of triosephosphate isomerase (32, 33), phosphoglycerate kinase deficiency (34) and phosphofructokinase deficiency (35).

### Congenital Dyserythropoietic Anemias

Congenital dyserythropoietic anemias (CDA) comprise a group of rare/very rare diseases characterized by ineffective erythropoiesis and morphological abnormalities of bone marrow erythroblasts (36, 37), caused by different molecular mechanisms affecting cell maturation and division. Three major types and other more rare or sporadic variants can be classified, on the basis of the typical morphology and on the affected genes (38–40). CDA type I, caused by biallelic mutations in *CDAN1* (CDAIa) or *c15orf41* (CDAIb) genes, is characterized by the presence of 2-5% binucleated erythroblasts of different size and shape in bone marrow, chromatin bridges between nuclei, and dense heterochromatin with a “Swiss cheese” appearance when observed at electron microscopy (41). CDA type II (CDAII) is a recessive disease caused by mutations in the *SEC23B* gene (42, 43), characterized by 10–35% binucleated and multinucleated erythroblasts which present with a peripheral double membrane, and hypoglycosylation of band 3 as a biochemical hallmark. CDAIII is caused by the dominant P916R mutation of *KIF23* gene with large multinucleated erythroblasts (44), whereas E325K mutation of *KLF1* gene is responsible for CDAIV (45) and mutations in *GATA1* gene cause an X-linked sporadic form (46).

## The Role of Naturally Occurring Antibodies in CHAs

**Figure 1:**
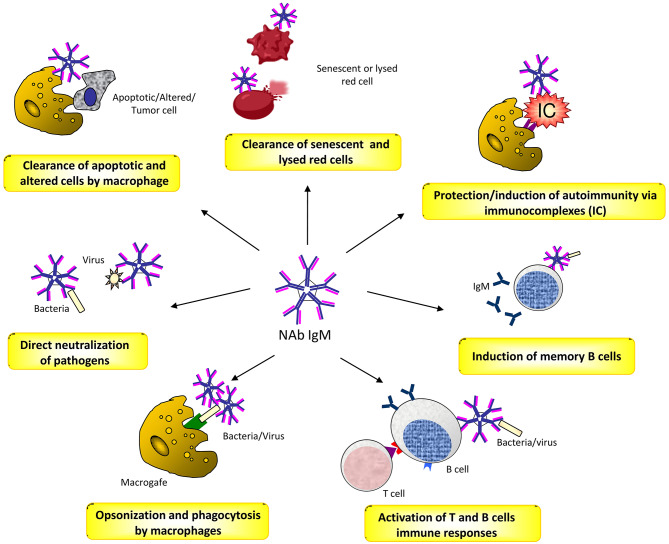
The several roles of Naturally Occurring Antibodies (NAbs).

Natural antibodies (Nabs) are circulating antibodies that, in healthy subjects, occur without known immune exposure or vaccination. They are mainly moderate affinity polyreactive IgM and are secreted by B1 cells, a subset of B cells that have been identified as CD20^+^CD27^+^CD43^+^ memory B cells without activation markers (47, 48). NAbs play different roles in health and disease (49). They contribute as a first line of defense from infection of bacterial, viruses, protozoa, and fungi (50, 51). This activity is mediated by opsonisation and neutralization of the pathogens, by activation of T cell and B cell responses, and by the induction of long-term immune memory cells (52, 53). NAbs play also a crucial role in the maintenance of the immune homeostasis by recognizing apoptotic cells membrane markers and promoting the process of their phagocytic clearance (54). Moreover, there is evidence that NAbs binding to inflammatory cytokines protect against improper inflammation (55). In addition, they concur to the opsonisation and removal of potentially harmful elements, thus exerting a physiologic antitumor surveillance (56). Finally, NAbs are closely related to autoimmunity, acting in a dual manner. On one hand, they prevent autoimmune disease by binding to immune-complexes and promoting their removal, or to self-antigens by increasing their exposure to immature B cells, and thus inducing tolerance (57, 58). On the other hand, in systemic autoimmune diseases, NAbs can bind to different self-molecules, such as nucleic acids, phospholipids, erythrocytes, serum proteins and cellular components, and cause disease through the formation of immune complexes (49, 59, 60) (Figure 1).

Regarding CHAs, NAbs anti-spectrin and anti-band 3 had been described long ago in sera from healthy subjects and in β-thalassemia patients, hypothesizing a physiologic role in the clearance of debris of lysed cells (61, 62). In particular, Reliene et al. (63) demonstrated the presence of high-affinity NAbs directed against RBCs (up to 140 molecules per cell) in band 3-deficient HS patients. Moreover, their number increased in with cell age, suggesting a possible role in removal of senescent cells. In line with these results, Zaninoni et al. (56) found that 61% of HS cases showed RBC-bound IgG positive values (up to 330 ng/mL), detected by mitogen-stimulated direct antiglobulin test (MS-DAT). The latter, is a sensitive test that may amplify autoantibody secretion, including NAbs production, through mitogen stimulation *in vitro* (64, 65). RBC-bound IgG were directed against different membrane proteins (α- and β-spectrin, band 3, and dematin) and were more evident in aged samples obtained after several days of storage at 4°C (56). As shown in Table 2, positive HS cases, mainly spectrin-deficient cases, had an increased number of spherocytes and showed a more hemolytic pattern (increased number of reticulocytes, unconjugated bilirubin and LDH values), suggesting that these antibodies may have a pathogenic role, participating in the loss of membrane area and reduction of surface-to-volume ratio (66). This phenomenon is less evident in splenectomised patients who lack the main organ in which RBC clearance occurs. In fact, the amount of RBC-bound IgG is slightly lower in these patients compared to non-splenectomised ones. Although there is no direct evidence that RBC antibodies induced by mitogen stimulation are NAbs, their increase with RBC age, and their greater amount in more hemolytic HS subjects are in favor of this hypothesis. Altogether these findings suggest that a humoral immune response has a role in removing senescent and damaged HS cells, thus participating in the clinical picture, and severity of the disease.

**Table 2:** Hematological characteristics of HS patients divided according to MS-DAT positivity.

	**MSDAT negative**	**MSDAT positive**
**Non-splenectomized**		
N° patients	30	48
Hemoglobin (g/dL)	12.5 ± 2.2	12.3 ± 1.8
Spherocytes (%)	5 (2–24)	7 (1–68)
Reticulocytes × 10^3^/mmc	147 ± 98	278 ± 133
Unconjugated bilirubin	2.7 ± 2.3	3.0 ± 2.1
LDH (U/L)	396 ± 158	473 ± 163
Haptoglobin <20 mg/dL (N)	22/30 (73%)	43/48 (89%)
IgG bound to RBC (ng/mL)	105 ± 31	331 ± 217
**Splenectomized**		
N° patients	6	3
Hemoglobin (g/dL)	15 ± 0.65	13.9 ± 1.6
Spherocytes (%)	8 (5–11)	4 (3–20)
Reticulocytes × 10^3^/mmc	85 ± 29	95 ± 77
Unconjugated bilirubin	0.5 ± 0.17	1.5 ± 1.8
LDH (U/L)	342 ± 54	322 ± 93
Haptoglobin <20 mg/dL (N)	0/6 (0%)	2/7 (35%)
IgG bound to RBC (ng/mL)	100 ± 44	277 ± 131

*Values are expressed as median (range) or mean±DS. Normal values are Hb: 13.6–16.7 g/dL; MCV 78-99 fL; reticulocytes: 16–84 × 10^3^/mmc; unconjugated bilirubin <0.75 mg/dL, aptoglobin: 30–200 mg/dL; LDH: 135–214 U/L. Data obtained from Zaninoni et al. (56)*.

## The Role of the Spleen and Effects of Splenectomy in CHAs

It is well-established that the spleen is the main catheretic organ involved in the removal of damaged or abnormal red blood cells, mainly via the macrophage system. In fact, splenectomy has been suggested as a possible therapeutic approach for various hemolytic diseases including CHAs. Its efficacy greatly varies among different pathologies, being maximal in HS, moderate in red cell enzyme defects, and minimal in dyserythropoietic anemias. Autoimmune hemolytic anemia (AIHA) and immune thrombocytopenia (ITP) are two acquired autoimmune disorders were splenectomy has been the only second line therapy for many years (67, 68). In recent years, the percentage of patients with CHAs or autoimmune cytopenias subjected to this therapeutic option has progressively declined due to the availability of new drugs and to the increasing awareness of possible complications. They include short- and long-term infections by encapsulated microorganisms (*Streptococcus pneumonie, Neisseria meningitides, and Hemophilus influenza)* (69), thrombotic events and pulmonary hypertension (70, 71). The mechanism underlying thrombotic complications are poorly understood. Early thrombotic events have been related to stasis in the splenic vein remnant, increased numbers of platelets, and to large spleen size previous surgery. Additional mechanisms, under investigation, are endothelial alterations, presence of activated platelets, and increased amounts of circulating procoagulant microvescicles. Moreover, there is an interplay between the coagulation and the immune system, particularly with the complement cascade as highlighted in paroxysmal nocturnal hemoglobinuria (72).

Concerning CHAs, a large retrospective analysis reported that splenectomy has been performed in 21% of HS patients and induced a median Hb increase of 3 g/dL (from 10.8 g/dL to 13.9 g/dL) (73). After splenectomy no infectious complications have been reported in a recent meta-analysis (74). On the contrary, episodes of stroke, pulmonary emboli or pulmonary arterial hypertension have been described with an overall risk 5.6-fold higher than in non-splenectomised (71, 75–77).

Regarding PKD, Zanella et al. (21, 22) reported that 18/61 (30%) patients had been splenectomised with an amelioration of the hemoglobin levels (median Hb increase of 1.8 g/dL, range 0.4–3.4). In the more recent and larger international series of splenectomised PKD cases, Grace et al. (23) showed that 59% of patients have been splenectomised at a median age of 6.5 years (range 0.4–37.8) with a median Hb increase of 1.6 g/dL. Sepsis and thrombotic events have been registered in 7 and 8%, respectively.

Regarding HSt, splenectomy is contraindicated in both dehydrated and overhydrated types, due to the highly increased risk of thromboembolic complications. In old case reports, severe thrombotic complications after surgery have been documented in 100% of cases, of which 3 were fatal (78–80). More recently, Andolfo et al. (81) reported that Hb levels did not improve and severe thrombotic episodes occurred in 5 *PIEZO1*-mutated splenectomized cases. Moreover, Picard et al. (82) described 12 cases in which splenectomy has been performed at a median age of 24 years (range, 4–41) and before the diagnosis of DHSt. Surgery didn't ameliorate hemolysis (mean Hb level 11.2±1.9 g/dL and reticulocytes count 280 ± 134 × 10^9^/L after splenectomy). Thrombotic complications occurred in all the 8 splenectomised patients with *PIEZO1* mutation, while in none of the 4 subjects with *KCNN4* mutation.

Finally, splenectomy has been described in 13/53 (25%) CDAI patients with severe anemia and mainly transfusion-dependent. Surgery has been performed mainly in adulthood (range 27–42 yrs) and 6/13 patients became transfusions-independent. However, the long follow-up performed revealed that 3 patients died, 1 of pulmonary arterial hypertension and 2 of overwhelming sepsis (83, 84). Concerning CDAII, Heimpel et al. (85) described that splenectomy has been performed in 22/48 (46%) patients at a median age of 19.9 years (range 1–50) with a median Hb improvement of about 1 g/dL, remaining below the reference values. The analysis of a larger series of 101 CDAII patients from 91 families, with a median follow-up of 23 years (range 0–65), is in line with these results (86): 40/ 101 cases underwent splenectomy, 16 of whom before diagnosis of CDAII, and at median age of 19 years (range 3–56). The rate of splenectomy dropped from 40 to 24% by considering only patients splenectomised after the diagnosis of CDAII, and further decreased to 7.5% by considering only the patients splenectomised in the past 15 years. The median Hb increase was 1.7 g/dL (range 1–6,7 g/dL, and splenectomy abrogated transfusion requirement in all patients but three. Information on splenectomy complications was available in 12 patients: one child had a thromboembolic event soon after surgery, and two patients had sepsis after 3 and 15 years. Table 3 summarizes available studies on splenectomy in CHAs with the relative efficacy and safety data.

**Table 3:** Effect of splenectomy in congenital hemolytic anemias.

	**Main hematological findings**	**Complications**	**References**
Hereditary Spherocytosis	Median Hb increase of 3 g/dL Normalization of reticulocytosis Decrease of unconjugated bilirubin and LDH levels	No infectious complications Thrombotic events (risk 5.6-fold higher)	(75) (76) (73) (77) (71) (74)
Dehidrated Hereditary Stomatoytosis (*PIEZO1*)	Hb amelioration in few reported cases No amelioration of Hb levels	Severe/fatal thrombotic complications (PHT, PE; priapism) Severe thrombotic events	(78) (79) (80) (81) (82)
Gardos Chanellopathy (*KCNN4*)	Amelioration of Hb levels	No thrombotic events	(81) (82)
Piryuvate Kinase deficiency	Median Hb increase of 1.6–1.8 g/dL Decrease of unconjugated bilirubin Reduction in the number of patients receiving regular transfusions	Sepsis in 7% of cases Thrombotic events in 8% of cases	(21) (22) (23)
Congenital Dyserythropoietic anemia type I	Amelioration of Hb levels Transfusion-independency in some cases	Fatal complications: 1 pulmonary arterial hypertension and 2 overwhelming sepsis	(83) (84)
Congenital Dyserythropoietic anemia type II	Hemoglobin concentration improved in all patients but remaining below reference values Decrease of bilirubin levels Median Hb increase of 1.7 g/dL Transfusion-independency in almost all cases	No infectious or thrombotic episodes Sepsis: 2 episodes Thrombotic event: 1 episode	(85) (86)

*PE, pulmonary embolism; PHT, pulmonary hypertension*.

In conclusion, the efficacy of splenectomy in CHAs mainly resides in the removal of the catheretic organ. However, as the spleen plays an important role in the immune system, this therapeutic option may be accompanied by a reduction of the immune competence with possible serious consequences. To reduce their development, patient education and immediate interventions in case of febrile episodes are pivotal. Additional important prophylaxis includes continuous antimicrobial therapy and periodical re-vaccination.

## Cytokine and Erythropoietin Levels in CHAs

It is well-known that there is a complex interplay between hemolysis, inflammation, and erythropoiesis (87). Although intravascular hemolysis is not the main pathogenic mechanism in CHAs, it may occur in acute and severe hemolytic crisis, and results in the release of cell-free hemoglobin that has pro-inflammatory properties (88). Moreover, it is clearly demonstrated that anemia of chronic inflammatory disease is driven by alterations of several immune-regulatory cytokines. In particular, overproduction of pro-inflammatory mediators, such as interleukin (IL)1-β, tumor necrosis factor (TNF)-α, IL-6, and interferon (INF)-γ, have been reported in several conditions including autoimmune diseases, chronic kidney and pulmonary disease, cancer, and chronic infections (89). In particular, in autoimmune hemolytic anemia several abnormalities of immune regulatory cytokines have been reported: high serum levels of IL-10 and IL-12 (90) and increased IL-1α, IL-2/IL-2R, IL-6, and IL-21. In cell supernatants, the T helper (Th)-1 cytokines IL-2 and IL-12 were reported elevated, whereas IFN-γ was found reduced, and the Th-2 cytokines IL-4 and IL-13 were increased, together with elevated production of IL-6, IL-10, and IL-17 (64, 91). Moreover, AIHA patients with active hemolysis showed further reduction of IFN-γ and increased secretion of transforming growth factor (TGF)-β that favor the differentiation of a Th-17 subset, which amplifies the pro-inflammatory and autoimmune response (92). It is known that inflammatory cytokines down-regulate erythropoietin (EPO) production, thus compromising erythropoiesis, and can activate erythrophagocytosis, especially in acute inflammation (89, 93).

**Figure 2:**
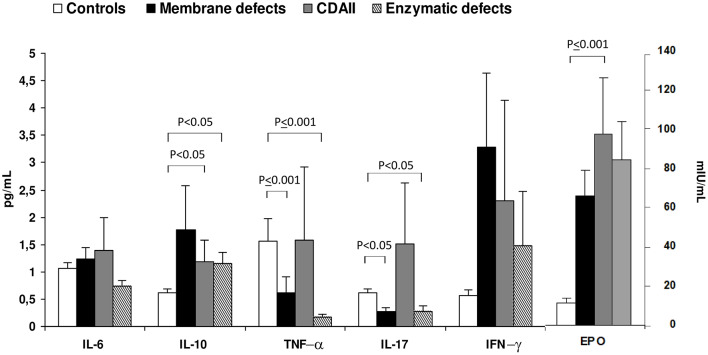
Cytokine and erythropoietin serum levels in congenital hemolytic anemias. Values are expressed as mean±SD. Data obtained from Barcellini et al. (94).

Regarding CHAs, little is known about cytokine levels. Barcellini et al. (94) described cytokine status and EPO levels in 52 patients with membrane or enzymatic defects and CDAII. As shown in Figure 2, IL-10 and IFN-γ were increased in all groups compared to age and sex matched controls, being particularly evident in membrane defects. IL-6 was increased as well, although to a lesser extent. Interestingly, CDAII and enzymatic defects showed a similar pattern regarding TNF-α and IL-17 with increased values of TNF-α and reduced levels of IL-17. Finally, EPO levels were increased in CHAs compared with controls, particularly in CDAII, possibly reflecting an attempt to compensate anemia. These alterations showed no relationship with severity of the clinical phenotype, i.e., degree of anemia and hemolysis. This was the first evaluation of cytokines in these diseases and results should be interpreted with caution due to the limited sample size and to the high inter-individual variation of the cytokine signature. However, it can be speculated that a chronic inflammation exists also in CHAs and may affect proper bone marrow compensatory erythropoiesis. Moreover, it may play a role in the complex interplay between hemolysis and iron overload.

## The Vicious Circle of Iron and the Immune System in CHAs

It is well-recognized that iron overload (IO) occurs in hemoglobinopathies, also because of transfusion support. Complication of IO are well-described in these diseases, and include cardiac dysfunction (arrhythmia, cardiomyopathy, hemosiderosis), liver cirrhosis, liver cancer and hepatitis, metabolism dysfunction (diabetes, hypogonadism, thyroid disorders, parathyroid, and less level of adrenal glands), and delays in sexual maturity, impotence and infertility (95–97).

Occurrence of iron overload is well-documented in dyserythropoietic anemias, PKD and HSt. Russo et al. (98) reviewed data of 205 CDAII showing that 57% patients had a serum ferritin level of >500 μg/ml, of whom 15% never transfused, and a transferrin saturation (TfSat) value of about 60%. More recently, IO was reported in 45% of PKD patients, as defined by ferritin levels >1,000 μg/L or chelation. A liver iron concentration (LIC) > 4 mg Fe/gdw was observed in 82% of patients by magnetic resonance imaging (MRI), even in absence of regular transfusions (23, 99). Moreover, van Strateen et al. (100), showed that LIC ≥ 3 mg Fe/gdw was present in 71% (31/44) of patients with CHAs and LIC ≥ 7 mg Fe/gdw was present in 36% (16/44), regardless of transfusion dependency and ferritin levels >1,000 μg/L. None of the patients had cardiac iron overload. Iron overload has been also described in HSt, particularly in the DHSt form and Gardos chanellopathy, where hyperferritinemia, high transferrin saturation or clinical iron overload are very frequent (81, 82, 101). In these cases hyperferritinemia is not related to transfusions since usually patients are not transfused on a regular basis. Serum ferritin level up to ~1,000 μg/L, TfSat value of about 60–70%, LIC ≥ 4 mg Fe/gdw, and cardiac T2^*^ <10 ms, have been described in case reports (102–105). Similar results have been reported in a large series of 126 patients were the mean±SD ferritin level at diagnosis was 764±480 μg/L (1,702 ± 1,048 μg/L in 5 *KCNN4* gene mutated cases, and 656 ± 428 μg/L in 40 *PIEZO1* mutated patients); mean liver iron content, evaluated by MRI, was 200 ± 103 μmol/g at diagnosis (82). Finally, Barcellini et al. (94) studied 52 patients with different CHAs showing that 60% of subjects had ferritin values> 500 μg/L. TfSat was > 50% in 31% of patients with membrane defects, in 66% with CDAII, and in 53% with enzymopathies. Moreover, non-transferrin-bound serum iron (NTBI) serum levels were increased in CDAII and moderately augmented in enzymatic defects. By MRI, median LIC value was 3.4 mg Fe/gdw (range 1.4–16.1) and 40% of patients, almost all CDAII, had a LIC ≥4 mg Fe/gdw (Table 4).

**Table 4:** Iron overload in congenital haemolytic anemias.

	**Main hematological findings**	**Complications**	**Reference**
Hereditary Spherocytosis	Median ferritin value 634 μg/L (192–1,171) (*n* = 26) Median TfSat value 36 % (23–67) Median NTBI value −0.08 μmol/L (−1.1 to 4.05) Mean ± SD hepcidin value 29.7 ± 4 ng/mL	LIC > 4 mg Fe/gdw in 8/26 cases 1 patient showed moderate cardiac IO (T2* 13 ms)	(94)
Dehidrated Hereditary Stomatoytosis (*PIEZO1*)	Ferritin value up to ~1,000 μg/L TfSat value of ~60–70%	LIC ≥ 4 mg Fe/gdw Cardiac IO with T2* <10 ms	(102) (103) (105) (104)
	Mean ± SD ferritin value 656 ± 428 μg/L (*n* = 40)	Mean liver iron content, evaluated by MRI, was 200 ± 103 μmol/g	(82)
	Median ferritin value 425 μg/L (*n* = 20)	Not reported	(81) (106)
Gardos Chanellopathy (*KCNN4*)	Mean ± SD ferritin value 1,702 ± 1,048 μg/L (*n* = 5)	Mean liver iron content, evaluated by MRI, was 200 ± 103 μmol/g	(82)
Piruvate Kinase Deficiency	Median ferritin value 425 μg/L (182–1,605) (*n* = 17) Median TfSat value 52% (22–89) Median NTBI value 0.26 μmol/L (−0.48 to 2.37), Mean ± SD hepcidin value 15.15 ± 3 ng/mL.	LIC > 4 mg Fe/gdw in 6/17 cases. Overall prevalence of IO was 45% (82/181) as defined by ferritin or chelation; 82% (67/82) as define by LIC > 4 mg Fe/gdw	(94)
	Median ferritin value 583 ng/mL (17–5,630).	7% (5/75) of cases had cardiac IO	(99) (23)
Congenital Dyserythropoietic Anemia type II	Median ferritin value 441 μg/L (206–1,605) (*n* = 9) Median TfSat value 85% (13–92) Median NTBI value 1.07 μmol/L (0.9–2.15) Mean ± SD hepcidin value 17.6 ± 6.5 ng/mL.	LIC > 4 mg Fe/gdw in 7/9 cases 1 patient showed moderate cardiac IO (T2* 12.7 ms)	(94)
	Median ferritin value 464.8 ± 55.9 μg/L (*n* = 109) Median TfSat value of ~60%	Not reported	(98)
	Median max ferritin value 668 μg/L (27–5,267) (*n* = 98) Median max TfSat value of 81% /20–157) (*n* = 79)	Not reported	(86)

*n, number of patients; TfSat, Transferrin saturation; NTBI, Non-transferrin-bound serum iron; LIC, liver iron concentration*.

**Figure 3:**
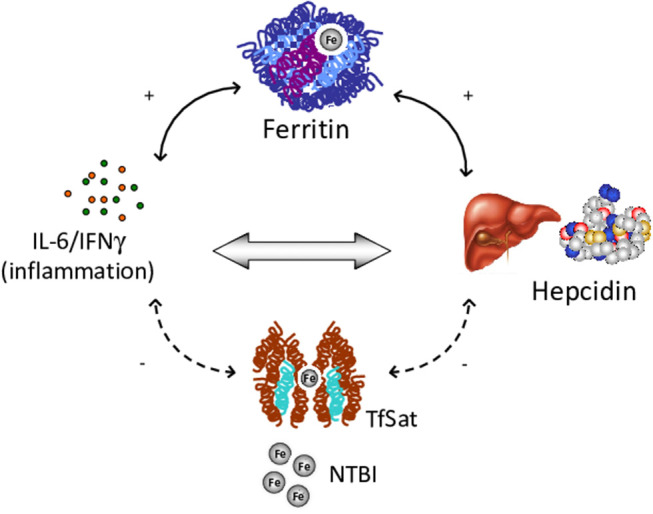
Vicious circle among chronic hemolysis, inflammatory response and iron overload. IL, Interleukin; IFN, Interferon; TfSat, Transferrin saturation; NTBI, Non-transferrin-bound serum iron.

Among factors possibly involved in IO, low hepcidin levels, ineffective erythropoiesis and an altered pro-inflammatory cytokine profile have been suggested to play different roles in CHAs (107–109). Ineffective erythropoiesis is probably the leading mechanism, since the greater frequency of IO is observed in CDAII, and dehydrated stomatocytosis (13, 110). In line with this hypothesis a correlation was observed between LIC and EPO levels (94). In the same series, hepcidin, the main hormone involved in the regulation of iron homeostasis, was slightly increased in CHAs compared with controls, and positively correlated with ferritin. Moreover, hepcidn positively correlated with the inflammatory cytokines IL-6 and IFN-γ, and has a direct pro-inflammatory activity (89, 93). Further evidence for the interplay between iron and inflammation comes from studies in DHSt, where hepcidin levels were decreased and erythroferrone (ERFE), the negative regulator of hepcidin, slightly increased. In patients with gain-of-function mutations in *PIEZO1*, inhibition of the bone morphogenetic proteins (BMP)/small mother against decapentaplegic (SMADs) pathway was involved in hepatic iron metabolism impairment (106). In addition, another important factor for iron balance is emojuvelin (HJV), a co-receptor of BMP that is degraded in juvenile hemochromatosis, causing severe hepcidin deficiency and iron overload. Fillebeen et al. (111) showed that HJV knocked-out mice failed to mount an appropriate hypoferremic response to acute inflammation caused by lipopolysaccharide. Finally, it is well-known that hemolytic crisis in CHAs may be triggered by infectious episodes that may therefore fuel the inflammatory loop. Overall the results suggest the existence of a vicious circle between chronic hemolysis, inflammatory response and IO (Figure 3).

## Conclusion

Although few data are reported on the role of the immune system in CHAs, several immune-mediated mechanisms are certainly involved in the pathogenesis of these rare diseases, namely naturally-occurring autoantibodies, spleen catheresis, overexpression of inflammatory cytokines, and iron overload. Regarding the first, naturally-occurring autoantibodies have a function in the opsonization of damaged/senescent erythrocytes and consequently further increase of their removal in the spleen, participating in the clinical picture and severity of the disease. Furthermore, splenectomy is performed in CHAs with variable degree of efficacy related to reduction of erythrocyte catheresis. However, it is important to remind that spleen is part of the immune system and its removal is associated with a variable immune deficiency, infections, and a higher thrombotic risk. Regarding the third mechanism, there is undoubtedly a role for pro-inflammatory cytokines in perpetuating chronic inflammation, which in turn may affect proper bone marrow compensatory erythropoiesis. This may account for a vicious circle among low-grade chronic inflammation, chronic hemolysis, and increased production of hepcidin, resulting in iron overload in a considerable and underestimated proportion of CHAs.

## Author Contributions

AZ, EF, WB, and PB prepared the manuscript. CV and AM critically revised the manuscript. All authors contributed to the article and approved the submitted version.

## Conflict of Interest

The authors declare that the research was conducted in the absence of any commercial or financial relationships that could be construed as a potential conflict of interest.
